# Ultrafiltration rate is an important determinant of microcirculatory alterations during chronic renal replacement therapy

**DOI:** 10.1186/s12882-017-0483-z

**Published:** 2017-02-20

**Authors:** Gerke Veenstra, Andrius Pranskunas, Inga Skarupskiene, Vidas Pilvinis, Marc H. Hemmelder, Can Ince, E. Christiaan Boerma

**Affiliations:** 1 Medical Center Leeuwarden, P.O. Box 888, Leeuwarden, 8934 AD The Netherlands; 2 Lithuanian University of Health Sciences, Kaunas, Lithuania; 3 Translational Physiology, Academic Medical Center, Amsterdam, The Netherlands; 4 Erasmus MC University Hospital Rotterdam, Rotterdam, The Netherlands

**Keywords:** Microcirculation, Hemodialysis, Microvascular alterations, Ultrafiltration rate, Negative fluid balance

## Abstract

**Background:**

Hemodialysis (HD) with ultrafiltration (UF) in chronic renal replacement therapy is associated with hemodynamic instability, morbidity and mortality. Sublingual Sidestream Dark Field (SDF) imaging during HD revealed reductions in microcirculatory blood flow (MFI). This study aims to determine underlying mechanisms.

**Methods:**

The study was performed in the Medical Centre Leeuwarden and the Lithuanian University of Health Sciences. Patients underwent 4-h HD session with linear UF. Nine patients were subject to combinations of HD and UF: 4 h of HD followed by 1 h isolated UF and 4 h HD with blood-volume-monitoring based UF. Primary endpoint: difference in MFI before and after intervention. During all sessions monitoring included blood pressure, heartrate and SDF-imaging. Trial registration number: NCT01396980.

**Results:**

Baseline characteristics were not different between the two centres as within the HD/UF modalities. MFI was not different before and after HD with UF. Total UF did not differ between modalities. Median MFI decreased significantly during isolated UF [2.8 (2.5–2.9) to 2.5 (2.2–2.8), *p* = 0.03]. Baseline MFI of each UF session was correlated with MFI after the intervention (*r*_s_ = 0.52, *p* = 0.006).

**Conclusion:**

During HD with UF or isolated HD we observed no changes in MFI. This indicates that non-flow mediated mechanisms are of unimportance. During isolated UF we observed a reduction in MFI in conjunction with a negative intravascular fluid balance. The correlation between MFI before and after intervention suggests that volume status at baseline is a factor in microvascular alterations. In conclusion we observed a significant decrease of sublingual MFI, related to UF rate during chronic renal replacement therapy.

## Background

Intermittent hemodialysis (HD) with concomitant ultrafiltration (UF) in chronic renal replacement therapy is associated with hemodynamic instability, usually referred to as ‘intradialytic hypotension’. The incidence of this phenomenon ranges between 30 and 90% depending on the definition on clinically relevant intradialytic hypotension. This unfavourable condition is not only associated with the inability to extract fluids adequately, but also with increased all-cause mortality, hospitalization for heart failure/volume overload and major adverse cardiac events [1–4]. In addition, intradialytic hypotension is likely to represent the tip of the iceberg with respect to consequences of changes in organ perfusion during HD. A striking discordance between hemodialysis-related symptoms or changes in (relative) blood volume and intradialytic hypotension has been reported [5, 6]. Intradialytic hypotension is more likely to represent a late symptom of a pre-existing gradual reduction in blood flow during HD, compensated by an increase in vascular resistance and cardiac performance. However, pre-existent cardiac morbidity and concomitant treatment is likely to disturb this compensation mechanisms. More importantly, decreased left ventricular compliance as a result of increased heart mass and a rapidly descending systemic vascular resistance are both risk factors for a decreased cardiac output and potentially hypotension in dialysis patients [7]. Apart from non-circulatory effects of HD this discordance also represents a fundamental problem within the current clinical assumption that blood pressure is directly related to organ perfusion. To overcome this problem direct visualisation and quantification of the sublingual microcirculation with a hand-held device has been suggested by Bemelmans and co-workers, as a non-invasive tool to trace ‘organ’ perfusion during HD [8]. Direct in-vivo microscopy of the sublingual area with sidestream dark field (SDF) imaging during HD revealed marked reductions in microcirculatory blood flow in the absence of intradialytic hypotension in the vast majority of patients. Despite the potential of these observations many questions remain to be answered. The incidence, aetiology and clinical relevance of the microvascular alterations remain to be elucidated. This study has 2 major objectives: 1. Are we able to reproduce previous observations in a comparable subset of patients; and 2. Are the observed microcirculatory alterations the result of UF, HD or a combination?

## Methods

### Study design and setting

Phase I consisted of a multi-centre prospective observational study conducted between October 2011 and December 2012. Participating centres were the Medical Centre Leeuwarden, a tertiary teaching hospital in the Netherlands and The Hospital of Lithuanian University of Health Sciences, an academic medical centre in Kaunas, Lithuania. Local ethical committees of both hospitals approved the study and written informed consent was obtained from every patient, according to applicable laws. In phase II (2012) of the study all patients included in the Netherlands were additionally subject to a single-centre prospective interventional study to compare different combinations of HD and UF rates. Study design was registered in advance at Clinicaltrials.gov (NCT01396980).

### Intervention

In phase I all patients were subject to their routine 4-h HD session, using a standard bicarbonate dialysate on normal temperature. During this period linear UF was maintained at a constant rate, in order to achieve a quantitative ultrafiltrate target, based upon the registered ideal dry weight of the patient. Primary endpoint is the difference in MFI between baseline and post intervention.

**Fig. 1:**
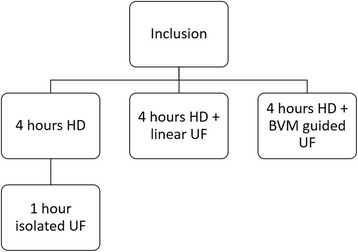
Study design. HD hemodialysis, UF ultrafiltration, BVM blood volume monitoring

In phase II patients from the Netherlands were subject to 2 additional combinations of HD and UF: 4 h of HD alone followed by 1 h isolated UF, and 4 h HD plus UF based on blood-volume-monitoring (BVM) (5008 hemodialysis machine®, Fresenius Medical Care) (Fig. 1) [9]. Sessions were assigned to each patient in random order and performed on the same day of the week; every patient served as his/her own reference. Primary endpoint is the difference in MFI between the HD/UF modalities post intervention.

### Measurements

During all sessions standard hemodynamic monitoring included blood pressure, heartrate and peripheral oxygen saturation using pulse oximetry. Sublingual in-vivo microscopy with sidestream dark field (SDF)-imaging, incorporated in a small hand-held camera, was performed in all patients at baseline and at the end of each session. For each timeframe 3 steady images of at least 10 s were obtained and recorded on digital videotape (SONY videowalkman GV-D 1000E®, Sony, Tokyo, Japan). Subsequent analysis was performed off-line and in random order with AVA software (Microvision Medical, Amsterdam, the Netherlands) [10]. Quantification of parameters of red blood cell velocity and capillary density was performed in accordance with an international consensus paper [11]. In short, red blood cell velocity in small vessels (<20 μm) is scored semi-quantitatively for each quadrant between 0 (stand still) and 3 (continuous normal flow) [12]. The average score of 3 × 4 quadrants is expressed as microvascular flow index (MFI). Total vessel density (TVD), as a determinant of capillary density, is calculated as the surface area of small vessels per mm^2^. Percentage of perfused vessels (PPV) is expressed as the percentage of perfused capillaries (MFI 2 and 3) divided by the total number of capillaries that crosses a grid of three horizontal and vertical equidistant lines. In phase II body composition monitoring (BCM; Fresenius Medical, Bad Homburg, Germany), based upon bioelectrical impedance analysis, and measurements of N-terminal pro b-type natriuretic peptide (NT-proBNP) and Troponine T were additionally performed prior to and after each intervention [13, 14].

### Statistics

All data are expressed as median [IQR]. Statistical analysis was performed with the Statistical Package for Social Sciences (SPSS 21, Chicago Illinois, USA). Due to the small sample size non-parametric tests for independent and paired data, as well as for correlation coefficients were applicable. A *p*-value < 0.05 was considered statistically significant. Based upon previous publications a sample size of 8 patients for phase II was considered adequate to detect a difference of 0.7 arbitrary units in MFI between the different HD/UF modalities [8].

## Results

### Phase I

During a 15-months period in 2011–2012 28 patients were included in the study. Overall, baseline characteristics were not significantly different between the 2 centres (Table 1). The primary endpoint MFI was not different before and after HD in combination with linear UF of 2.3 [1.3–3.2] l over a 4 h period (Table 2). In addition, hemodynamic variables did not change over time, with the exception of a small, but significant increase in peripheral oxygen saturation (Table 2).

**Table 1:** Baseline characteristics phase I and II

Variables	All (*n* = 28)	LT (*n* = 19)	NL (*n* = 9)	*p*-value
Men, %	57	42	89	0.04
Age, years	64 [53–74]	60 [49–70]	69 [55–78]	0.29
Years on HD	3 [1–6]	3 [1–7]	3 [1–5]	0.94
Remaining diuresis, l/24 h	0.2 [0–0.5]	0.3 [0–0.6]	0.2 [0–0.4]	0.60
Weight, kg	78 [67–87]	73 [66–79]	85 [88–92]	0.03
BMI, kg/m2	26 [24–28]	27 [23–30]	26 [25–28]	0.94
UF volume, l	2.3 [1.3–3.2]	2.6 [1.6–3.3]	1.7 [1.2–2.1]	0.10
Cause of ESRD, %				
Diabetes	32	21	56	
Hypertension	18	26	0	
ADPKD	7	5	11	0.27
ATN	14	16	11	
Miscellaneous	29	32	22	
Drugs, %				
ß-blocker	61	42	67	1.0
ACE inhibitor	61	74	33	0.1
Calcium antagonist	47	58	22	0.09

*LT* Lithuania, *NL* Netherlands, *HD* hemodialysis, *BMI* body mass index, *UF* ultrafiltration, *ESRD* end stage renal disease, *ADKPD* autosomal dominant polycystic kidney disease, *ATN* acute tubular necrosis, *ACE* angiotensin converting enzyme

**Table 2:** Results phase I (*n* = 28). Hemodynamic and microcirculatory variables of small vessels (<20 μm) before and after hemodialysis in combination with linear ultrafiltration

Variables	Baseline	Post HD/UF	*p*-value
Mean arterial pressure, mmHg	93 [76–111]	96 [84–110]	0.39
Heartrate, beats/min	69 [62–80]	73 [60–84]	0.24
SpO_2_, %	97 [96–98]	98 [98–99]	0.009
MFI, AU	3 [2.8–3]	3 [2.8–3]	0.55
TVD, mm/mm^2^	22.2 [18–29.8]	22.7 [19.9–29]	0.11
PPV, %	98 [96–100]	98 [96–99]	0.35

*HD* hemodialysis, *UF* ultrafiltration, *SpO*_*2*_ peripheral oxygen saturation, *MFI* microvascular flow index, *AU* arbitrary units, *TVD* total vessel density, *PVD* perfused vessel density, *PPV* percentage of perfused vessel

### Phase II

**Fig. 2:**
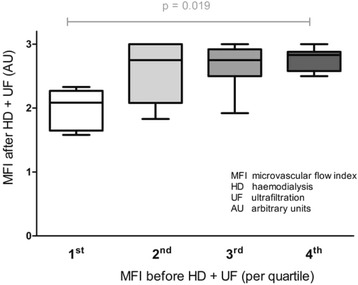
Distribution of post-intervention microvascular alterations per quartile of pre-intervention microvascular blood flow. *P*-value across groups

In a 2-month period in 2012 all 9 patients from the Netherlands that participated in phase I were included. There was no statistical difference between baseline characteristics of different HD/UF modalities (Table 3). Total UF did not differ between HD/UF modalities, with the exception of HD alone. However, UF rate was significantly higher in isolated UF (*p* < 0.001) in comparison to combined HD/UF modalities (Table 3). Median MFI decreased significantly during isolated UF [2.8 (2.5–2.9) to 2.5 (2.2–2.8), *p* = 0.03], but remained unaltered during the other HD/UF modalities. We observed no significant difference between HD + linear UF and HD + BVM-guide UF (Fig. 2). With the exception of isolated HD, BCM-derived overhydration and NT-pro-BNP decreased significantly during all HD/UF modalities, indicating a similar trend in volume status.

**Table 3:** Results phase II (*n* = 9). Laboratory data, microcirculatory variables of small vessels (<20 μm) and bioelectrical impedance analysis before and after intervention

Variables	Baseline	Post-intervention	*p*-value
UF isolated			
UF	-	1.7 [1.2–2]	
UF rate, l/h	-	1.7 [1.2–2]^†^	
MFI, AU	2.8 [2.5–2.9]	2.5 [2.2–2.8]	0.03
TVD, mm/mm^2^	17.7 [16.5–18.4]	18.7 [16.1–20.3]	0.26
Hematocrit, %	37 [35–39]	39 [36–44]	0.11
NT-pro-BNP, pmol/l	599 [215–1702]	580 [158–1440]	0.01
Troponine T, ng/l	90 [60–150]	87 [56–130]	0.08
BCM overhydration, l	1.4 [0.6–3.2]	0.3 [−0.1–0.8]	0.02
HD isolated			
UF	–	0 [0–0]	
UF rate, l/h	–	0 [0–0]^†^	
MFI, AU	2.8 [2.5–2.9]	2.8 [2.7–2.9]	0.61
TVD, mm/mm^2^	17.7 [16.5–18.4]	18.6 [14.6–19.7]	0.86
Hematocrit, %	37 [35–39]	NA	
NT-pro-BNP, pmol/l	599 [215–1702]	NA	
Troponine T, ng/l	90 [60–150]	NA	
BCM overhydration, l	1.4 [0.6–3.2]	NA	
HD + linear UF			
UF		1.7 [1.2–2.1]	
UF rate, l/h		0.42 [0.3–5.1]^†^	
MFI, AU	2.9 [2.5–3]	2.6 [2.2–2.9]	0.12
TVD, mm/mm^2^	17.8 [16.6–18.8]	19.8 [17.9–21.5]	0.07
Hematocrit, %	38 [35–40]	40 [35–41]	0.18
T-pro-BNP, pmol/l	618 [279–1926]	536 [167–1003]	0.01
Troponine T, ng/l	94 [61–179]	84 [62–139]	0.02
BCM overhydration, l	1.8 [0.5–5.3]	0.3 [−0.9–3.2]	0.02
HD + BVM-guided UF			
UF		2.0 [1.5–2.1]	
UF rate, l/h		0.5 [0.39–0.52]^†^	
MFI, AU	2.8 [2.5–3]	2.8 [1.9–2.9]	0.06
TVD, mm/mm^2^	18.8 [16.8–20.6]	18.2 [16.9–20.6]	0.77
Hematocrit, %	37 [34–39]	39 [35–42]	0.12
NT-pro-BNP, pmol/l	574 [229–2011]	511 [172–1163]	0.01
Troponine T, ng/l	81 [54–379]	79 [54–387]	0.12
BCM overhydration, l	2.4 [1.5–5.1]	0.2 [−0.9–4.2]	0.04

*UF* ultrafiltration, *HD* hemodialysis, *BVM* blood volume monitoring, *MFI* microvascular flow index, *TVD* total vessel density, *PPV* percentage of perfused vessels, *NT-pro-BNP* n-terminal pro b-type natriuretic peptide, *BCM* body composition monitoring ^†^ *p* < 0.001 across different HD/UF modalities

**Fig. 3:**
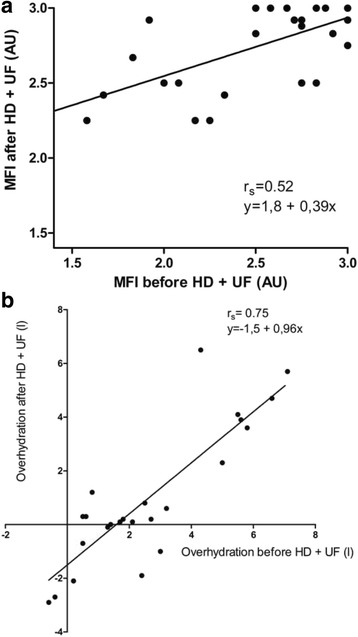
**a**. Correlation in microvascular alterations between pre- and post-intervention. **b**. Correlation in body-composition-monitoring-derived overhydration between pre- and post-intervention

Baseline MFI of each UF session (irrespective of UF modality) was significantly correlated with MFI after the intervention (*r*_s_ = 0.52, *p* = 0.006; Fig. 3a). The coefficient of correlation for pre- and post-intervention overhydration was also significant (*r*_s_ = 0.75, *p* < 0.001; Fig. 3b).

## Discussion

In phase I of the study we did not observe a reduction in sublingual microvascular blood flow or capillary density during HD in combination with linear UF. In an attempt to unravel the aetiology of previously observed alterations in microvascular blood flow during combined HD/UF we changed the ultrafiltration modalities in a subset of patients in phase II. During isolated HD we observed no changes in microvascular blood flow. This indicates that potential non-flow mediated mechanisms for microvascular derangements, such as hemodialysis-induced inflammation and hypercoagulation, are unlikely to play an important role [15, 16]. However, during isolated UF, a modality with the highest UF rate in which the influence of HD itself is absent, we were indeed able to reproduce the reduction in microvascular blood flow, as observed by others [8, 17]. This suggests that the observed reduction in microvascular blood flow may be the result of a negative intravascular fluid balance. In case the UF rate exceeds the ability to mobilize interstitial fluids towards the intravascular space, an increase in vascular resistance or viscosity causes a reduction in microvascular blood flow. The fact that Bemelmans et al. observed a marked attenuation of impaired microvascular blood flow during autotransfusion with a Trendelenburg manoeuvre after HD/UF is also in line with this aetiology [8].

An important issue is the question why we did not observe previously reported microcirculatory alterations during HD in combination with linear UF. At first glance there are important similarities with both articles: UF was 2.5 [1.6–3.5] l and 2.5 ± 0.88 l respectively, and over a similar period of time [8, 17]. But a closer look reveals a marked reduction in MFI at baseline in both studies; 2.8 [2.5–5] and 2.7 ± 0.5 versus 3 [2.8–3] in our study. Baseline values of these previous publications indicate pre-existing microvascular derangement prior to the start of HD/UF, since they are outside the range of healthy volunteers [18, 19]. A secondary analysis of our data in phase II revealed a significant correlation between MFI at baseline and MFI after the intervention, indicating that indeed volume status at baseline is an additional factor in the development of microvascular alterations during UF, irrespective of its modality. This suggest that our patients were less prone for microcirculatory changes, but that a higher rate of isolated ultrafiltration can result in impaired microperfusion in this group. Furthermore, our data suggest that 56% of the post-intervention overhydration is caused by pre-treatment overhydration (Fig. 3b).

Further studies are needed to investigate a potential correlation between ultrafiltration-derived changes in microvascular flow and morbidity and/or mortality in hemodialysis patients.

The limitations of the study are related to the small sample size in phase II. We anticipated a potential difference in MFI between the UF modalities, based upon previous observations. However, the observed changes in MFI were considerably smaller. As a consequence we may have been unable to detect an existing difference in sublingual microvascular blood flow between HD + linear UF and HD + BVM-guided UF (type I error).

## Conclusions

In conclusion we observed a significant decrease of sublingual microvascular blood flow due to rapid isolated ultrafiltration. Additional interventions with different combinations of HD and UF revealed that HD per se is not associated with changes in microvascular flow. During ultrafiltration over a longer period of time, and in combination with hemodialysis, baseline abnormalities were associated abnormal microvascular blood flow at the end of the renal replacement session. By design this study is not suitable to establish the clinical relevance of the observed microvascular alterations.

## Abbreviations

BVM: Blood-volume-monitoring
HD: Hemodialysis
MFI: Microcirculatory blood flow
NT-proBNP: N-terminal pro b-type natriuretic peptide
PPV: Percentage of perfused vessels
SDF: Sublingual Sidestream Dark Field
Trop-T: Troponine T
TVD: Total vessel density
UF: Ultrafiltration
